# Tracking the Main States of Dynamic Functional Connectivity in Resting State

**DOI:** 10.3389/fnins.2019.00685

**Published:** 2019-07-09

**Authors:** Qunjie Zhou, Lu Zhang, Jianfeng Feng, Chun-Yi Zac Lo

**Affiliations:** ^1^Shanghai Center for Mathematical Sciences, Fudan University, Shanghai, China; ^2^Institute of Science and Technology for Brain Inspired Intelligence, Fudan University, Shanghai, China; ^3^Oxford Centre for Computational Neuroscience, Oxford, United Kingdom; ^4^Department of Computer Science, University of Warwick, Coventry, United Kingdom

**Keywords:** community clustering, signed networks, modularity, temporal changes, resting state functional magnetic resonance image

## Abstract

Dynamical changes have recently been tracked in functional connectivity (FC) calculated from resting-state functional magnetic resonance imaging (R-fMRI), when a person is conscious but not carrying out a directed task during scanning. Diverse dynamical FC states (dFC) are believed to represent different internal states of the brain, in terms of brain-regional interactions. In this paper, we propose a novel protocol, the signed community clustering with the optimized modularity by two-step procedures, to track dynamical whole brain functional connectivity (dWFC) states. This protocol is assumption free without *a priori* threshold for the number of clusters. By applying our method on sliding window based dWFC’s with automated anatomical labeling 2 (AAL2), three main dWFC states were extracted from R-fMRI datasets in Human Connectome Project, that are independent on window size. Through extracting the FC features of these states, we found the functional links in state 1 (WFC-C_1_) mainly involved visual, somatomotor, attention and cerebellar (posterior lobe) modules. State 2 (WFC-C_2_) was similar to WFC-C_1_, but more FC’s linking limbic, default mode, and frontoparietal modules and less linking the cerebellum, sensory and attention modules. State 3 had more FC’s linking default mode, limbic, and cerebellum, compared to WFC-C_1_ and WFC-C_2_. With tests of robustness and stability, our work provides a solid, hypothesis-free tool to detect dWFC states for the possibility of tracking rapid dynamical change in FCs among large data sets.

## Introduction

Spontaneous fluctuations are a fundamental mechanism representing neural signals that has been largely explained by functional magnetic resonance imaging (fMRI) data. Resting-state functional connectivity (FC) can demonstrate the intrinsic network organizations of human brain (Friston, 2011). The cognitive activities of high order brain function involve the dynamic interplay of a set of brain circuits rather than a specific region, and the spontaneous activity in rest is also predictive of task and behavior performance (De la Iglesia-Vaya et al., 2013; Reineberg et al., 2015; Tavor et al., 2016). Accumulating studies have proposed to detect the spatiotemporal organization of dynamic functional connectivity (dFC), and of dynamic whole-brain functional connectivity (dWFC) (Calhoun et al., 2014; Kopell et al., 2014; Preti et al., 2017), showing how brain FC organized over time.

Clustering analysis, particularly k-means is one of the most common methods of categorizing dFC patterns (Calhoun et al., 2014). It partitions n samples in observation space into k clusters, where each sample belongs to the nearest cluster according to its distance from the cluster centroid. However, it requires the pre-defined number of clusters k and is sensitive to initial values that may lead to different results. Hierarchy clustering (HC) aims to building a dendrogram which represents a hierarchy of cluster, and the samples could be attributed to a sub-cluster within a main cluster. Thus, HC is a more flexible method to understand the dFC structures in different levels (Vidaurre et al., 2017). However, it also requires the definition of a specific threshold for cluster separation. Both k-means and HC are not assumption free and need *a priori* knowledge for categorization of the states of brain activity.

The selection of the number of clusters or the threshold may bias or affect the interpretation of the states while lacking comprehensive understanding of the underlying mechanism of dFC. Principal component analysis (PCA) coverts a number of possibly correlated variables into a set of linearly uncorrelated variables, called principal components. It has been used to investigate dynamic brain connectivity patterns, “eigenconnectivities,” by ranking and extracting the principal components of dWFC’s with higher variability across time and subjects (Leonardi et al., 2013). Though PCA is a powerful tool to detect the different features of dFC, it needs to bear a risk of information loss during the reduction of dimensionality. Other state detection models based on hidden Markov chain also require prior knowledge of the expression form and the number of states (Robinson et al., 2015; Ryali et al., 2016; Vidaurre et al., 2017).

These approaches are able to uncover the similar time-varying recurring connectivity patterns into states, and have revealed the characteristics of dFC linking with the human demographic characterization, cognitive behaviors and diseases (Baker et al., 2014; Calhoun et al., 2014; Karahanoğlu and Van De Ville, 2015; Zhang et al., 2016). However, heterogeneities are widely observed across studies. There is still a lack of reliable methods for the research of dFC networks. In this study, we focused on the co-variation of FCs over time by detecting the state for dWFC’s across subjects and time from Human Connectome Project (HCP) data. We proposed the modularity-optimized community clustering algorithm to categorize the dWFC’s in an unsupervised and data-driven fashion. This can provide a more appropriate clustering method while little is known in dWFC’s states. As the computation for community clustering is computationally expensive and time-consuming, we also proposed a two-steps clustering process to reduce the cost of our proposed algorithm.

## Materials and Methods

### Participants and Data Acquirements

#### HCP

The dataset used for this study was collected from HCP^1^ (WU-Minn Consortium). Our sample includes 812 subjects (ages 22–35 years-old, 450 females) scanned on a 3T Siemens connectome-Skyra scanner. For each subject, a three-dimensional T1 structural image was acquired at 0.7 mm isotropic resolution with 3D MPRAGE acquisition. The four blood-oxygen-level dependent (BOLD) resting state fMRI (R-fMRI) runs were acquired in separate sessions on two different days, each for approximately 15 min (2 mm× 2 mm× 2 mm spatial resolution, TR = 0.72 s, 1200 timepoints, multiband acceleration factor of 8, with eyes open and relaxed fixation on a projected bright cross-hair on a dark background). The WU-Minn HCP Consortium obtained full informed consent from all participants, and research procedures and ethical guidelines were followed in accordance with the Institutional Review Boards (IRB) of Washington University in St. Louis, MO, United States (IRB #20120436). To identify WFC, the whole brain was parcellated into 120 regions according to the automated anatomical labeling (AAL2) atlas (Rolls et al., 2015), with names and abbreviations listed in Table 1.

**Table 1:** The anatomical regions defined in each hemisphere and their label in the automated anatomical labeling atlas 2 (AAL2, Rolls et al., 2015).

ID	Region description	AAL2	Lobe	Abbreviation
1, 2	Precentral gyrus	Precentral	Sensorimotor	PreCG
3, 4	Superior frontal gyrus, dorsolateral	Frontal_Sup	Frontal	SFG
5, 6	Middle frontal gyrus	Frontal_Mid	Frontal	MFG
7, 8	Inferior frontal gyrus, opercular part	Frontal_Inf_Oper	Frontal	IFGoperc
9, 10	Inferior frontal gyrus, triangular part	Frontal_Inf_Tri	Frontal	IFGtriang
11, 12	IFG pars orbitalis	Frontal_Inf_Orb	Frontal	IFGorb
13, 14	Rolandic operculum	Rolandic_Oper	Frontal	ROL
15, 16	Supplementary motor area	Supp_Motor_Area	Sensorimotor	SMA
17, 18	Olfactory cortex	Olfactory	Frontal	OLF
19, 20	Superior frontal gyrus, medial	Frontal_Sup_Med	Frontal	SFGmedial
21, 22	Superior frontal gyrus, medial orbital	Frontal_Med_Orb	Frontal	PFCventmed
23, 24	Gyrus rectus	Rectus	Frontal	REC
25, 26	Medial orbital gyrus	OFCmed	Frontal	OFCmed
27, 28	Anterior orbital gyrus	OFCant	Frontal	OFCant
29, 30	Posterior orbital gyrus	OFCpost	Frontal	OFCpost
31, 32	Lateral orbital gyrus	OFClat	Frontal	OFClat
33, 34	Insula	Insula	Subcortical	INS
35, 36	Anterior cingulate & paracingulate gyri	Cingulate_Ant	Frontal	ACC
37, 38	Middle cingulate & paracingulate gyri	Cingulate_Mid	Frontal	MCC
39, 40	Posterior cingulate gyrus	Cingulate_Post	Parietal	PCC
41, 42	Hippocampus	Hippocampus	Temporal	HIP
43, 44	Parahippocampal gyrus	ParaHippocampal	Temporal	PHG
45, 46	Amygdala	Amygdala	Subcortical	AMYG
47, 48	Calcarine fissure and surrounding cortex	Calcarine	Occipital	CAL
49, 50	Cuneus	Cuneus	Occipital	CUN
51, 52	Lingual gyrus	Lingual	Occipital	LING
53, 54	Superior occipital gyrus	Occipital_Sup	Occipital	SOG
55, 56	Middle occipital gyrus	Occipital_Mid	Occipital	MOG
57, 58	Inferior occipital gyrus	Occipital_Inf	Occipital	IOG
59, 60	Fusiform gyrus	Fusiform	Temporal	FFG
61, 62	Postcentral gyrus	Postcentral	Sensorimotor	PoCG
63, 64	Superior parietal gyrus	Parietal_Sup	Parietal	SPG
65, 66	Inferior parietal gyrus, excluding supramarginal and angular gyri	Parietal_Inf	Parietal	IPG
67, 68	SupraMarginal gyrus	SupraMarginal	Parietal	SMG
69, 70	Angular gyrus	Angular	Parietal	ANG
71, 72	Precuneus	Precuneus	Parietal	PCUN
73, 74	Paracentral lobule	Paracentral_Lobule	Parietal	PCL
75, 76	Caudate nucleus	Caudate	Subcortical	CAU
77, 78	Lenticular nucleus, Putamen	Putamen	Subcortical	PUT
79, 80	Lenticular nucleus, Pallidum	Pallidum	Subcortical	PAL
81, 82	Thalamus	Thalamus	Subcortical	THA
83, 84	Heschl’s gyrus	Heschl	Temporal	HES
85, 86	Superior temporal gyrus	Temporal_Sup	Temporal	STG
87, 88	Temporal pole: superior temporal gyrus	Temporal_Pole_Sup	Temporal	TPOsup
89, 90	Middle temporal gyrus	Temporal_Mid	Temporal	MTG
91, 92	Temporal pole: middle temporal gyrus	Temporal_Pole_Mid	Temporal	TPOmid
93, 94	Inferior temporal gyrus	Temporal_Inf	Temporal	ITG
95, 96	Cerebellum Crus I	Cerebelum_Crus1_L	Cerebellum	CRBLCrus1
97, 98	Cerebellum Crus II	Cerebelum_Crus2_L	Cerebellum	CRBLCrus2
99, 100	Cerebellum lobule III, hemisphere	Cerebelum_3_L	Cerebellum	CRBL3
101, 102	Cerebellum lobule IV V, hemisphere	Cerebelum_4_5_L	Cerebellum	CRBL45
103, 104	Cerebellum lobule VI, hemisphere	Cerebelum_6_L	Cerebellum	CRBL6
105, 106	Cerebellum lobule VII b, hemisphere	Cerebelum_7b_L	Cerebellum	CRBL7b
107, 108	Cerebellum lobule VIII, hemisphere	Cerebelum_8_L	Cerebellum	CRBL8
109, 110	Cerebellum lobule IX, hemisphere	Cerebelum_9_L	Cerebellum	CRBL9
111, 112	Cerebellum lobule X, hemisphere	Cerebelum_10_L	Cerebellum	CRBL10
113	Cerebellum lobule I II, vermis	Vermis_1_2	Cerebellum	Vermis12
114	Cerebellum lobule III, vermis	Vermis_3	Cerebellum	Vermis3
115	Cerebellum lobule IV V, vermis	Vermis_4_5	Cerebellum	Vermis45
116	Cerebellum lobule VI, vermis	Vermis_6	Cerebellum	Vermis6
117	Cerebellum lobule VII b, vermis	Vermis_7	Cerebellum	Vermis7
118	Cerebellum lobule VIII, vermis	Vermis_8	Cerebellum	Vermis8
119	Cerebellum lobule IX, vermis	Vermis_9	Cerebellum	Vermis9
120	Cerebellum lobule X, vermis	Vermis_10	Cerebellum	Vermis10

*Column five provides a set of abbreviations for the anatomical descriptions.*

### Data Preprocessing

#### HCP Data

The minimally preprocessed R-fMRI data were used, conducted by HCP Functional Pipeline v2.0 (Glasser et al., 2013), including gradient distortion correction, head motion correction, image distortion correction, and spatial transformation to the Montreal Neurological Institute (MNI) space, with one step spline resampling from the original functional images. The linear trend and quadratic term were removed from these functional images, and several nuisance signals were regressed from the time course of each voxel using multiple linear regression, including cerebrospinal fluid, white matter, and Friston 24 head motion parameters. Finally, temporal band-pass filtering (0.01–0.1 Hz) was performed to reduce the influence of low-frequency drift and the high-frequency physiological noise. The preprocessed time-courses were used for further functional connectivity analyses.

### Sliding Window Based Dynamic Functional Connectivity

**FIGURE 1:**
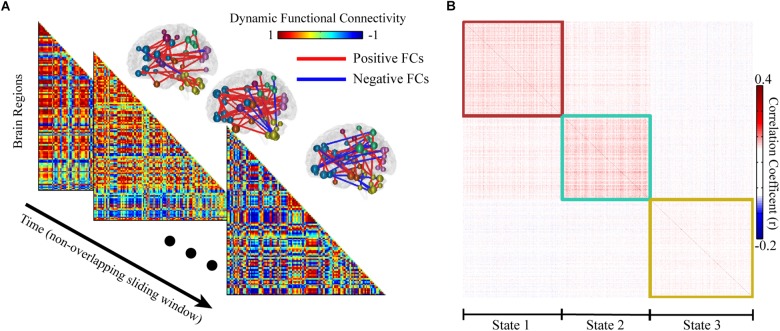
Sliding window based whole functional connectivity (WFC). **(A)** The whole brain dynamic functional connectivity matrix was computed with 14.4 s non-overlapping sliding window (length of 20 time points). The corresponding top 100 significant FCs are shown for illustration at upper right of the matrix. **(B)** An example of the community clustering results across time and subjects. The similar (reoccurred) network patterns were clustered into 3 modules, representing 3 states. The similarity of dynamic functional connectivity was defined as their Pearson correlation coefficient.

Either voxel or regional based BOLD signals can be used to calculate FCs. Here, we considered to process the regional based BOLD signals without losing any generality. We denoted time series {*x_i_*(*t*), *t* = 0, 1, ⋯, *N*, *i* = 0, 1, ⋯, *M*}, where *t* is time and *i* is the brain region. To characterize the dynamics of FCs, all BOLD signals were segmented into *T* non-overlapped sliding window with length *L* (Figure 1A). Fisher-z transformed Pearson correlations between all pairs of regional BOLD signal were calculated and normalized in each window as following.

$${\mathrm{FC}}_{\mathrm{ij}}(s)≜\frac{\mathrm{FZ}\left(r_{\mathrm{ij}}(s)\right)-\mu (s)}{\sigma (s)}$$

*r_ij_* (*s*) is the Pearson correlation between subset of signals *x_i_* (*t_s_*) and *x_j_* (*t_s_*) where *t_s_* = *s*, *s* + 1, ⋯, *s* + *L* − 1, and FZ(⋅) is the Fisher r-z transform

$$\mathrm{FZ}\left(r_{\mathrm{ij}}(s)\right)≜\frac{1}{2}\ln\left(\frac{1+r_{\mathrm{ij}}(s)}{1+r_{\mathrm{ij}}(s)}\right)$$

μ (*s*) and σ (*s*) represent the mean and standard deviation of the total $\frac{M(M-1)}{2}$ different pairs of FZ(*r_ij_* (*s*)), separately. Therefore, we obtained *N*/*L* dWFC’s networks for each subject. Because of the expensive computation, we used a two-steps clustering process to reduce the cost of the clustering algorithm. The dWFC’s calculated from all the time windows of each subject are grouped into sub-datasets for community clustering. The similarity matrix was presented by the Pearson correlation coefficient between any pair of dynamic dWFC’s for further states detection.

### Community Detection of Signed Graph

Each dWFC is considered as a vertex in graph theory. The community clustering algorithm assigns a graph with n vertices into *c* communities σ_*i*_ ∈ {1, 2, …, *c*}; i.e., each node was assigned to a community σ_*i*_, where *i* = 1, 2, …, *n*. Q-modularity of a weighted graph is defined as the edge weights within the community minus the expected edge weights of them (Leicht and Newman, 2008); i.e., Q = $\frac{1}{m}$ ∑_*i,j*_ (*A_i,j_* − *p_i,j_*) δ_*i,j*_, where δ_*i,j*_ = 1 if σ_*i*_ = σ_*j*_ and 0 otherwise; *p_i,j_* = *k_i_k_j_*/*m* represents the expected edge weight between *i* and vertex *j*; *m* is total the weight of all vertexes. *A* is adjacent matrix, where *A_i,j_* is the exact edge weight between vertex *i* and vertex *j*. By maximization of the Q-modularity, the community structure is determined with dense connections as an intra-community feature, while the sparse connections as inter-community features. As declared above, it is natural to use the similarity matrix, calculated from Pearson correlation coefficient of all pairs of dWFC’s, as the adjacent matrix in community clustering. In this study, the adjacent matrix *A* is a signed weighted matrix, and we employ an approach based on an extended signed Q-modularity of the graph (Lu et al., 2017). The graph is divided into two graphs composed by positive edges and negative edges, respectively, represent by *A*^+^ and *A*^−^, where $A_{i,j}^{+}$ ≥ 0 and $A_{i,j}^{-}$ ≤ 0. The extended signed Q-modularity equals (Lu et al., 2017): (i) the fraction of edge weights, of which both head and tail nodes fall within the same community, minus (ii) the expected value of the edge weights of a random graph that follows the same positive weight degree distribution of the intrinsic graph, plus (iii) the expected value of the edge weights of a random graph that follows the same negative weight degree distribution of this intrinsic graph. This can be formulated as

$$\text{Q}=\frac{1}{2m}\sum_{i,j}(A_{i,j}-p_{i,j}^{+}+p_{i.j}^{-}){\;\delta }_{i,j}$$

**FIGURE 2:**
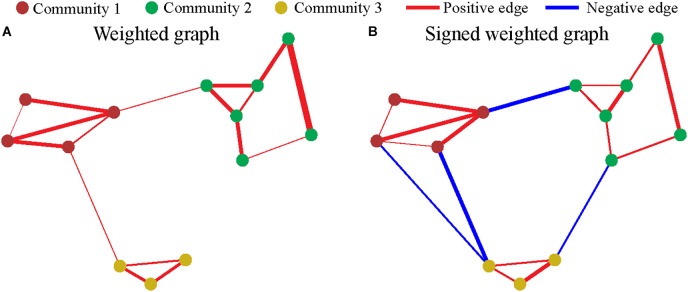
Community in graph. Each dot represents a vertex (node), and the color of nodes represent the community. Each line represents an edge, and the width and color represent the weighting and sign, respectively. **(A)** The community of weighted graph. **(B)** The community of signed weighted graph.

*m* is the sum of the absolute values of elements of the matrix *A*. $p_{i,j}^{\pm }$ stands for the expected coupling probability between vertex *i* and vertex *j* based on positive and negative coupling, respectively, represented by *A*^±^. Figure 2 illustrates the examples of the community structure in the “weighted” and “signed weighted” graph. The fast community detection algorithm (CDA) is used in maximize Q-modularity (Le Martelot and Hankin, 2013). The code from http://www.elemartelot.org/index.php/programming/cd-code was modified for handling of signed weighted edges.

### Two-Steps Community Clustering of dWFC’s for Large Data-Set

**FIGURE 3:**
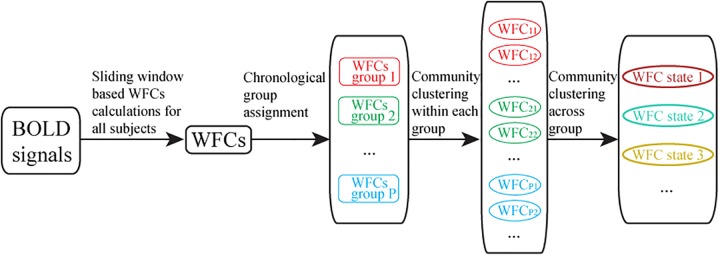
Flowchart of two-steps community clustering of dynamic whole brain function connectivity. (1) The extraction of dynamic whole brain functional connectivity based on sliding window; (2) Random group assignment for community clustering, where each group consists of a number of dWFC’s from all subject; (3) Community clustering results within each group, and the cluster centroids (averaged dWFC of the same state in each group) were preserved; (4) Final community clustering for the cluster centroids obtained from groups.

**FIGURE 4:**
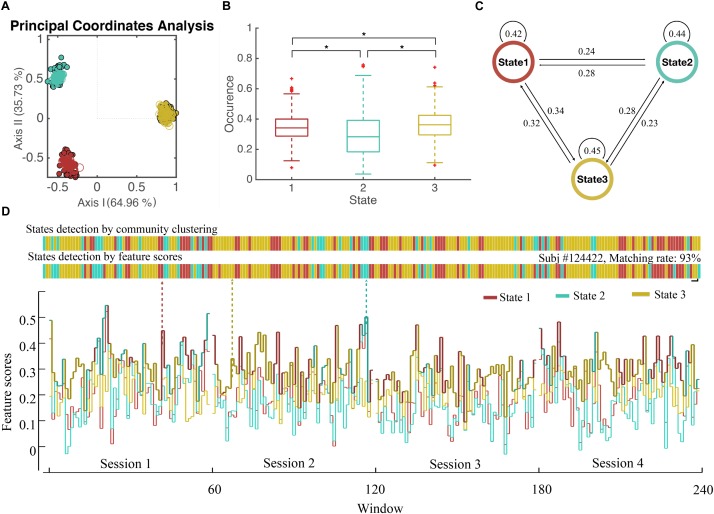
State Detection of Dynamical WFC. **(A)** The scatter plot of WFC’s in principal coordinate analysis. Each point represents a cluster centroid (the averaged WFC’s of the same community) detected in step 1. (Dots represent centroids in chronological groups and circles represent random groups) Distance between WFC points is defined by 1-correlation_wfc-wfc_^2^, where principal coordinate analysis projects those WFC points into 2D spaces while preserved the original distance as much as possible. **(B)** The boxplot of the occurrence of three detected states for all subjects and the *p*-value of two-sample *t*-test between the occurrence of different states. **(C)** The transition rate between three detected states. **(D)** The states of dynamic functional connectivity for a single subject (subject #124422 as an example) were detected based on individual community clustering and feature scores. The states show in colors according to the WFC communities in **(A)**.

The correlation coefficient for each pair of dWFC’s for an individual subject was computed as the similarity index for community detection. Ideally the community detection was performed across all subjects and time. However, the computation is extremely high when the subject population is large. In consideration of reducing the memory footprint and calculation time, this stage was developed in two steps due to the large amount of dWFC’s (Figure 3). Firstly, all the dWFC’s were separated into many sub-groups in chronological order such that each subject assigned a number of dWFC’s to each given group, denoted by S. That is, there were S dWFC’s from each subject in each group. The number of groups equals to the total number of dWFC’s of each subject divided by the amount in each group, i.e., $\frac{N}{\mathrm{LS}}$. The clustering algorithm was applied separately in each group, and the cluster centroids (mean of dWFC’s within a cluster) were kept. Next, all the cluster centroids extracted from different groups could be further clustered by applying the community detection on the correlation matrix of cluster centroids. We also randomly selected $\frac{N}{\mathrm{LS}}$ samples from 194880 dWFC samples (60 windows×4 sessions×812 subjects) into a group for 100 times. To test the stability and similarity of the clustering results from each group, we compared the clustering centroids obtained in random groups with those obtained in chronological groups, regardless of its sampling method (Figure 4A). Finally, we used the Davies–Bouldin index (DBI) (Davies and Bouldin, 1979), a well-known clustering quality measure by averaging the maximal similarity between each cluster and all other clusters, as a metric for evaluating the clustering performance both in step 1 (for dWFC’s in each group) and step 2 (for centroids from different groups). The smaller the index is, the better the clustering result is. Furthermore, we also used k-means algorithm to compare the rationality of the number of states with our method. For each group, we fixed the number of clusters as K from 1 to 6, and set 100 different initial values to detect the best partition with the minimal intra-class distance.

### Detection of Connectivity States

After two step clustering, all of the dWFC’s were assigned to the corresponding communities, which we defined the “states” here, according to their cluster centroids. The occurrence, transition rate, and mean lifetime of these states were calculated as dynamic parameters for all WFC’s in MR sessions (Ryali et al., 2016). The features of corresponding WFC communities were computed by averaging all dynamic WFC’s from each community, denoted as WFC-C*_i_*. Here we define the “feature score” by computing the correlation coefficient between WFC-C*_i_* and a given WFC, and the highest feature score among the states could predict the corresponding state.

## Results

### The Three States of Dynamic Whole-Brain Functional Connectivity

We applied our method in R-fMRI data from 812 healthy adults released by HCP to estimate the functional network connectivity states. The AAL2 atlas was considered first so that the number of regions M was 120. The length of the time series N was 4800. We set *L* = 20 and the influence of window length had been illustrated in Supplementary Figure S1. We set *S* = 5 due to the large computation consumption and we finally obtained 48 groups (a larger S could help to reduce the inconsistency between groups, see Supplementary Figure S1). The WFC’s within a community follows a common variation trend (positive correlation, Figure 1B) while those from different communities do not, or even follow a reversed variation trend (negative correlation). Noted that cluster centroids extracted in step 1 are distinctly divided into three communities (Figure 4A), both for the chronological groups and random groups, which revealed high resemblance of clustering results between groups. Thus, each WFC in a given time window of a given subject could be assigned to one of the three WFC state. The feature of the corresponding WFC community (WFC-C) was calculated by averaging all dynamic WFC’s from each community; and we computed the feature score among the three WFC-C’s to represent the predicted state for the original WFC’s. The distribution of the matching rate between the clustering states and the predicted states was 93.3% on average (Figure 4D), which may be helpful to detect the state for an unknown network without performing the clustering. For dynamic parameters, the state 3 showed the highest occurrence, whereas the state 2 showed the lowest occurrence (Figure 4B). The transition between state 1 and state 3 showed the most frequent rate (Figure 4C). There was no difference in mean lifetime that the three states had an averaged mean lifetime of 24.8 s for state 1, 25.1 s for state 2, and 25.9 s for state 3.

### The Evaluation of the Number of States

**FIGURE 5:**
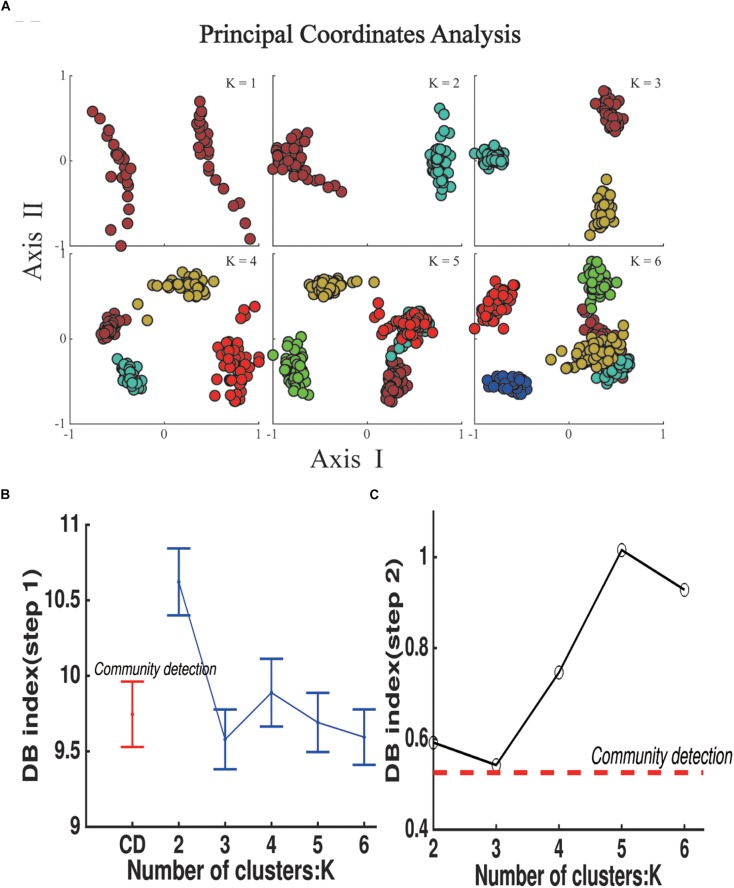
State Detection results using k-means clustering algorithm in step 1. **(A)** The scatter plot of k means centroids obtained in step 1 in principal coordinate analysis, K represents the number of the clusters in each group and N represents the number of communities detected by Modularity-based algorithm in step 2. **(B)** DB index for the clustering results for groups in step 1. Dots represent the mean value for 48 groups and error bars represent standard deviation. **(C)** DB index for the clustering results of k means centroids (blue polygon) and community centroids (red dash line).

The k-means algorithm was used to compare with our method (Figure 5A). The DBI was used to evaluate the clustering results of both steps. The mean DBI of 48 groups reached to the minimum of 9.58 while *K* = 3 in step 1 (Figure 5B), showing a better clustering result for small groups compared to CDA (the mean DBI = 9.74). However, the DBI of k-means centroids of *K* = 3 also achieved the optimal clustering performance in step 2 with the minimum of 0.54 (Figure 5C), whereas DBI of community centroids reached a smaller value of 0.52, a better result of overall clustering across groups. Both of the results in two steps indicated that the number of 3 clusters was the best for categorization of dWFC’s states.

### Features of the Whole-Brain Functional Connection States

**FIGURE 6:**
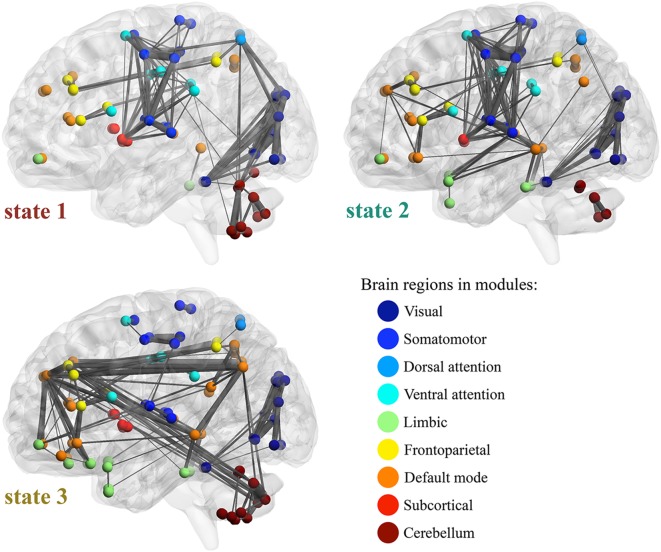
Feature of WFC states. Top 200 functional connections are illustrated in each WFC states, with the Yeo’s 7 functional modules, subcortical and cerebellar regions. The width of the connections represents the connectivity strength. The transition rates among states are indicated by the arrows. For state 1 (WFC-C1), the high FCs in mainly includes functional links both within and across visual, somatomotor, attention and cerebellar (posterior lobe) modules. WFC-C2 was similar with WFC-C1 in those high FCs, however, the FCs in WFC-C2 between cerebellum and the sensory and attention modules were decreased, and higher connections within and across limbic, default mode and frontoparietal modules, in which medial temporal gyrus (MTG), Superior temporal gyrus of temporal pole (TPOsup), inferior temporal gyrus (ITG), inferior parietal gyrus (IPG), dorsolateral superior frontal gyrus (SFG) and medial superior frontal gyrus (SFG medial) are highly involved. In WFC-C3, FCs within sensory and attention modules are still active, but FCs across those modules are decreased. Another feature of WFC-C3 high values of FCs in default network modules, as well as FCs across modules including default, limbic and cerebellum networks. MTG, precuneus (PCUN), angular gyrus (ANG), middle frontal gyrus (MFG), superior parietal gyrus (SPG), and Crus1/Crus2 in cerebellum are highly involved.

The AAL2 regions were assigned to Yeo’s seven functional modules according to the top ratio (the percentage of voxels of specific region within each network) (Yeo et al., 2011). Cerebellum and subcortical regions are added as two additional modules. Figure 6 illustrates the top 200 FC’s in the three WFC-C’s with functional modules, and the transition rates among states from HCP data. For state 1 (WFC-C_1_), the highest FC’s mainly include functional links both within and across visual, somatomotor, attention and cerebellar (posterior lobe) modules. The highest FC’s in WFC-C_2_ were similar with WFC-C, but FC’s linking limbic, default mode and frontoparietal modules were more involved whereas the cerebellum, sensory and attention modules were less involved. In WFC-C_3_, the FC’s linking default mode, limbic, and cerebellum were more involved, whereas somatomotor, dorsal, ventral attention, and visual modules were much less so, compared to WFC-C_1_ and WFC-C_2_.

### Robustness of dWFC’s States Across Window Lengths

The clustering result is independent of window length (Supplementary Figure S2), shown by the detection of dWFC’s states with various window lengths among 10, 20, 30, 40, and 50. The averaged dWFC’s in the same community had a high level of similarity that their Pearson correlation were close to 1, seen from the diagonal elements of each 3 × 3 matrix (Supplementary Figure S2B). Whereas, comparing the off-diagonal elements between correlation matrices of different window size, we still observed a trend that the differences between three averaged dWFC’s would reduce as the window length increased.

### The Influence of Parcellation Methods

We also detected three communities using dWFC’s calculated from two different additional atlases, the Shen-268 atlas (Shen et al., 2013) and Power 264 atlas (Power et al., 2011; Supplementary Figure S3). The results showed that the number of dynamic states was independent to the parcellation schemes. However, the detected state in each window was different across atlases. By matching the most overlapping states, the averaged matching rate of states extracted between the AAL2 Shen-268 was 82.7%, and the Power was 66.9%. Besides, we also randomly relocate the state sequence of the samples, the matching rate was significantly lower than the estimated matching rate (*p* < 0.0001), indicating that the states obtained across the atlases were similar but not identical.

## Discussion

We proposed a new method to categorize and track time-varying networks in R-fMRI studies. It involves two-step community detection, which is computing efficient and provides robust results in large data set application. In recent years, various methods were proposed to capture time-varying networks in R-fMRI studies (Pinotsis et al., 2013; Calhoun et al., 2014; Cavanna et al., 2017; Preti et al., 2017). Essentially, it involves two main considerations.

The first consideration, what is the best feature to represent the time-varying networks. For example, ICA could be used to reveal the spatial-temporal structure of the fMRI signals in either signal subject or group of subjects (Calhoun et al., 2009). Time and frequency decomposition of regional coherence was also calculated through cross wavelet transform (Yaesoubi et al., 2015). However, most common method is sliding window based FCs (Hutchison et al., 2013; Thompson, 2018; Reinen et al., 2018), as brain function are accomplished by the interplay of a set of brain areas rather than a specific region (De la Iglesia-Vaya et al., 2013). A recent study questioned the validity, stability and statistics significance for various dFC pattern detecting method (Hindriks et al., 2016). The optimal window size remains unknown. To track rapid temporal changes in FC, shorter window is necessary for high temporal resolution; while FC calculation requires longer window for robustness and statistics significance. It may need further studies to address this question through our method. We calculated FC the on various window sizes to test the reliability of the results. In our application on HCP data set with high tempo-spatial solution resolution, the three whole-brain dFC states are stable and independent of sessions and window lengths. The robust results suggest that our method could be helpful to establishing the golden standard in dWFC’s tracking in R-fMRI analysis (Shakil et al., 2016).

The second consideration is mainly a machine learning problem or a clustering problem. Lacking prior knowledge about the categorization of dynamical brain states, an unsupervised learning method especially clustering analysis is more suitable for detecting dynamical brain states. K-means, a prototype-based clustering method, is the most widely used in clustering analysis for its convenience and computing speed. However, it needs to set the number of states and initial values in advance and requires a relatively balanced data structure for good performance. Though many different methods have been applied, the number of states in the brain still remains unknown. For example, two brain states were revealed as a within-network state and a between-network state in both healthy and Parkinson disease patients (Kim et al., 2017). Di and Biswal (2015) separated out triple brain states: salience-, default-, and motor- networks. Seven brain states have also been discovered in work by Allen et al. (2014) through group ICA based-FC and k-means clustering. Further, as many as 13 clusters of innovation-driven co-activation patterns were detected in work (Karahanoğlu and Van De Ville, 2015). Another important issue of clustering analysis is the measure of distance or similarity between samples. Euclidean distance is an intuitive and commonly used distance. However, the Euclidean distance of WFC depends largely on the overall level of the functional connectivity, which could be affected by measurements, individual difference. The Pearson correlation induces a distance that remains unchanged and is equivalent to the Euclidean distance after normalization of the data. That is, 1 − *corr*(*x*,*y*) = ∥ *x* − *y* ∥^2^/2m, where *x* − *y* represents the Euclidean distance between sample x and y and m is the dimension of data. It measures the consistency of the sequence of FCs within the network between two WFC’s that will not be influenced by the overall functional connectivity value. Therefore, we used Pearson correlation to measure the similarity but also normalized each dynamical brain network for some following analysis of the detected brain states.

Due to the large sample size, we complete the clustering in two steps that we calculate the cluster centroids in each group and combine these results by clustering all the centroids. But it brings up a problem to select an appropriate state number K for each group and deal with the differences of results caused by different initial values. Hierarchical clustering can detect the hierarchical relationship in the data and it does not need to set the initial values. But it is more impossible to afford the large computation cost, because the computational complexity of hierarchical clustering is at least O(n^2^), which n represents the amount of dWFC. Moreover, the two-steps clustering strategy is not suitable for hierarchical clustering for it may break or disrupted the hierarchy of data when dividing samples into several groups and leave a tricky problem of matching samples at the same level from different groups. PCA helps to discover and describe different FC patterns through an appropriate number of PCs called “eigenconnectivities,” while it is still a question how to classify dWFC’s into different states so that we can track the dynamical changes of whole brain network structure. Hidden Markov chain based methods are usually performed directly on the bold signal time series of the brain rather than functional connectivity structure, they require a pre-given form of the probability distribution of each state as well as the number of states. Lacking a comprehensive understanding of the underlying mechanism of dWFC’s states, we categorize the dWFC’s through community detection methods based on the similarity of dWFC’s pair, working in an unsupervised, data-driven fashion. Finally, by computing the feature score between networks (the similarity), we can easily estimate an unknown state of network into a specific state, without redundant clustering procedures. This is helpful for further studies of the dynamic networks.

By comparison with k-means clustering, our proposed method with two-step of CDA showed better superiority. On the one hand, the DBI showed that the best number of clustering was 3 with k-means, indicating the CDA could detect the optimal number for the states of dWFC’s. On the other hand, compared to CDA, although k-means showed a smaller mean value of DBI for all groups in the first step, the larger DBI in the second step indicated longer distances between the centroids among groups, showing the weakness for the overall detection by the two step clustering method. According to the procedure of k-means algorithm, a possible reason might be that the k-means is sensitive to outliers (Hautamäki et al., 2005). When an outlier is added to a given cluster, the center of the cluster will move toward to the outlier, resulting in the change of the criteria to update the members of this cluster. Finally, the members of the cluster are more likely close to the outliers. In contrast, the community clustering showed better robustness than k-means. According to the fast community detection algorithm (Le Martelot and Hankin, 2013), an outlier alone can little influence the update of the community structure in each iteration because of its small degree, and therefore the community among groups had more stable and similar structures, showing its advantage for subdividing a large sample size into several groups of small samples.

By taking advantage of the higher temporal resolution in HCP, we can reduce the window to less than 10 s while maintaining sufficient samples to calculate correlation coefficients. However, it still remains unclear as to what the optimal window for detecting dynamical brain states is. Although community clustering methods are robust for summarizing the generality of dFC’s independent of time and subjects; this method might not be sensitive to individual heterogeneities. Further studies are needed to address whether there might be sub states within the three dFC states, i.e., to identify the hierarchical structure of the dynamic FC’s. The self-converged community clustering method to detect the connectivity states, does not rely on the appearance of a clear gap between any two individual dFC’s from various brain states (Leicht and Newman, 2008). It is a more appropriate clustering method while few are known in dWFC states. Besides, the two-steps community clustering protocol for large R-fMRI data sets is robust and computing efficient. A distinct gap between community centroids of different states, regardless of which groups they come from, showing that our method performed stably in each group. These results revealed that there were three states existed for all the dWFC across subjects and time, with the robustness with various window lengths and parcellations. Of note, the states of dWFC’s were not identical across parcellations, because of the location of regions, region size, including/excluding cerebellum, and the extracted time series were different, resulting in different dWFC’s across atlases. The parcellation scheme may affect the dWFC’s with specific functions (e.g., involving cerebellum or not), and the diversity of states among atlases may be further studied.

## Ethics Statement

The WU-Minn HCP Consortium obtained full informed consent from all participants, and research procedures and ethical guidelines were followed in accordance with the Institutional Review Boards (IRB) of Washington University in St. Louis, MO, United States (IRB #20120436).

## Author Contributions

LZ and CYL designed the research. QZ and LZ performed the research. QZ, LZ, and CYL analyzed the data. QZ, LZ, CYL, and JF wrote the manuscript.

## Conflict of Interest Statement

The authors declare that the research was conducted in the absence of any commercial or financial relationships that could be construed as a potential conflict of interest.

## Footnotes

1 http://www.humanconnectomeproject.org/
