# Supplementary material for: Tracking the Main States of Dynamic Functional Connectivity in Resting State
Source: Front Neurosci. 2019 Jul 9;13:685. doi: 10.3389/fnins.2019.00685 (PMC6629909; doi:10.3389/fnins.2019.00685)
Supplement: Supplementary file 1 [file Image_1.pdf]

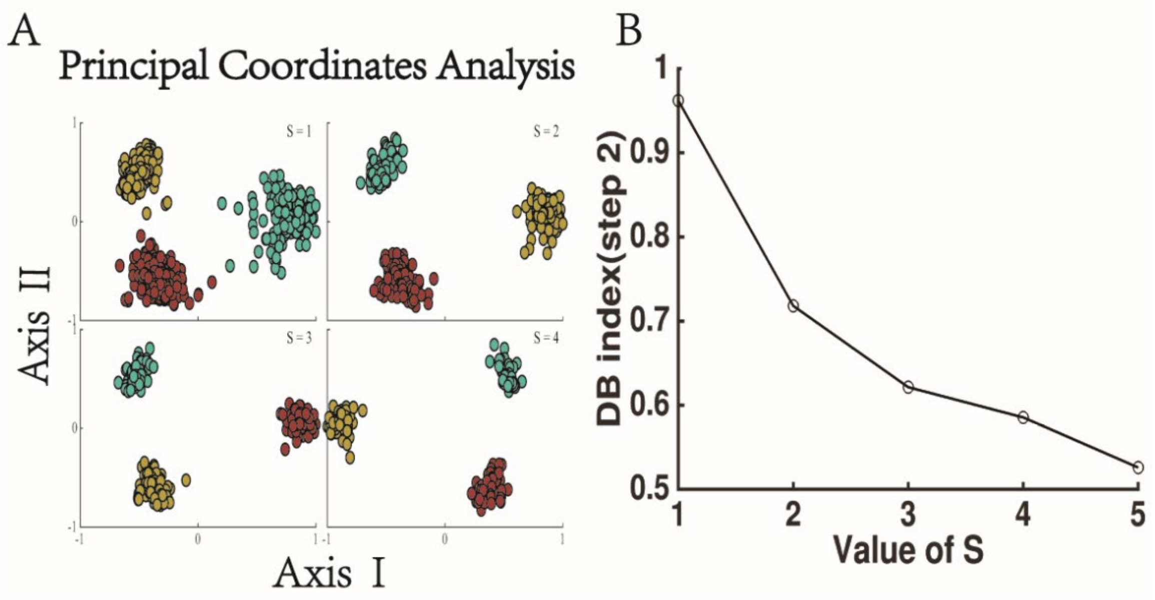

Figure S1. The Influence of the Amount of dWFC's in Each Group A. The scatter plot of cluster centroids for S=1 to 4. Each group had 812*S dWFC's which were chronologically selected from all subjects. **B.** Davies-Bouldin index was calculated according to the clustering result for S from 1 to 5.

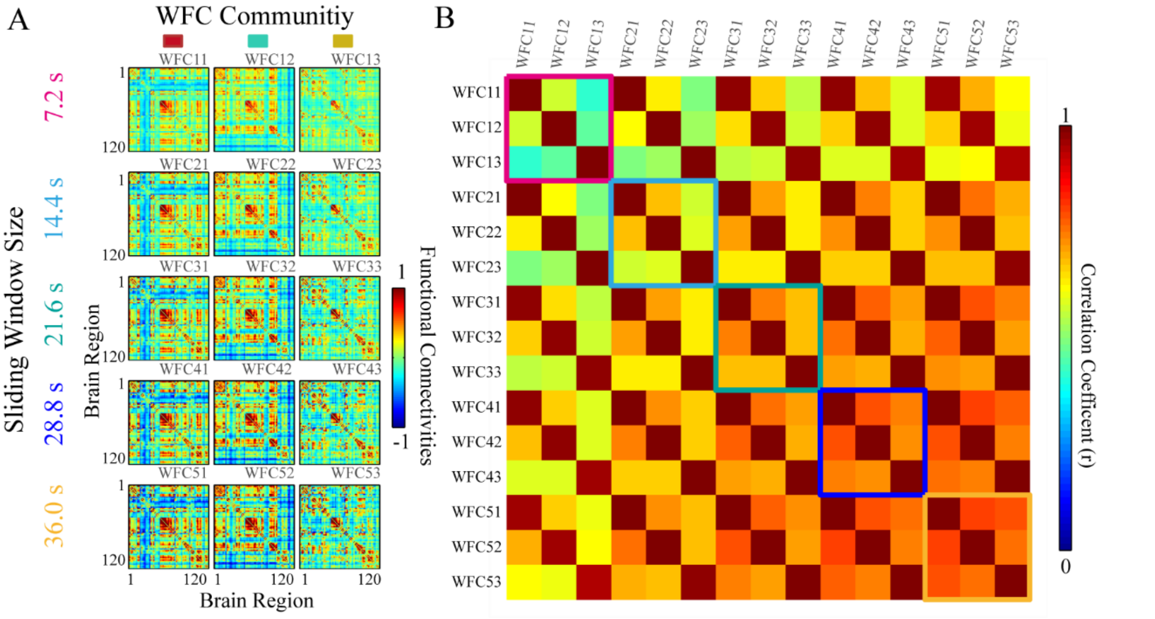

Figure S2. Community Clustering Feature of Dynamical Functional Connectivity Based on Different Sliding Window Size. A. Three D-WFC communities were detected based on five different sliding window size; averaged WFC communities were shown. **B.** Correlation matrix of all averaged

WFC's in A.

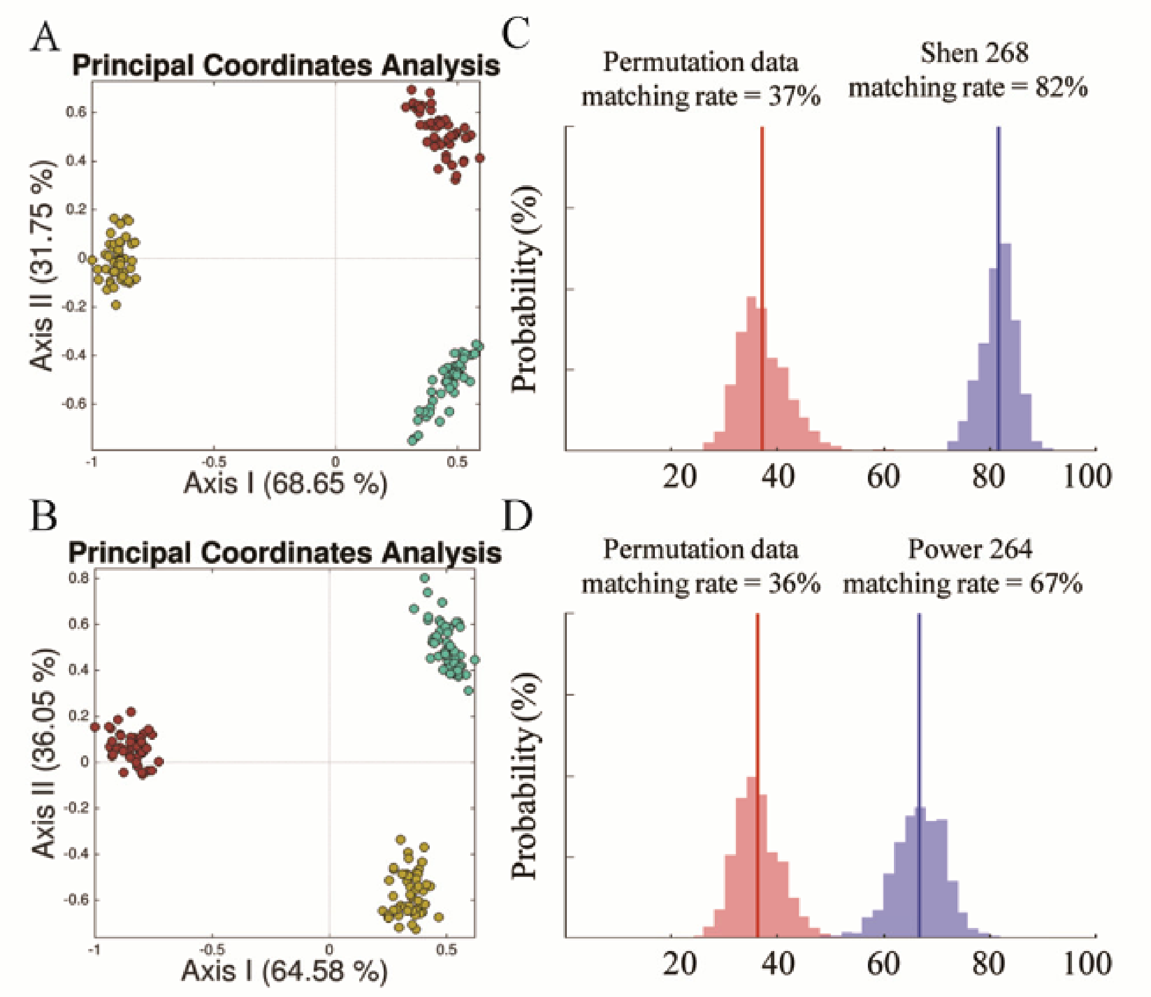

Figure S3. The Clustering and The State Detection Results Across Parcellations. The scatter plot of community clustering results of principle coordinates analyses based on Shen-268 atlas **(A)** and Power 264 coordinates **(B)**.The matching rate for 812 subjects between the states detected between AAL2 atlas and **(C)** Shen 268 atlas/ **(D)** Power 264 atlas. The blue histograms represent the distribution of the matched states between atlases, and the red histograms represent the distribution of the matching rate in 10000 permutation tests. detected. The order of states was adjusted according to maximum overlapping with AAL2 states.
